# Solulin reduces infarct volume and regulates gene-expression in transient middle cerebral artery occlusion in rats

**DOI:** 10.1186/1471-2202-12-113

**Published:** 2011-11-14

**Authors:** Yu-Mi Ryang, Jon Dang, Markus Kipp, Karl-Uwe Petersen, Astrid V Fahlenkamp, Jens Gempt, Dominik Wesp, Rolf Rossaint, Cordian Beyer, Mark Coburn

**Affiliations:** 1 Department of Neurosurgery, Klinikum rechts der Isar, Hospital of the Technical University Munich, Ismaningerstr. 22, 81675 Munich, Germany; 2 Department of Neurosurgery, University Hospital of the RWTH Aachen, Pauwelsstraße 30, 52074 Aachen, Germany; 3 Institute of Neuroanatomy, Faculty of Medicine, RWTH Aachen University, Wendlingweg 2, 52074 Aachen, Germany; 4 PAION Deutschland GmbH Aachen, Martinstr. 10-12, 52062 Aachen, Germany; 5 Department of Anesthesiology, University Hospital of the RWTH Aachen, Pauwelsstraße 30, 52074 Aachen, Germany; 6 Department of Neurosurgery, Georg-August-University Hospital, Robert-Koch-Straße 40, 37075 Göttingen, Germany

## Abstract

**Background:**

Thrombolysis after acute ischemic stroke has only proven to be beneficial in a subset of patients. The soluble recombinant analogue of human thrombomodulin, Solulin, was studied in an *in vivo *rat model of acute ischemic stroke.

**Methods:**

Male SD rats were subjected to 2 hrs of transient middle cerebral artery occlusion (tMCAO). Rats treated with Solulin intravenously shortly before reperfusion were compared to rats receiving normal saline i.v. with respect to infarct volumes, neurological deficits and mortality. Gene expression of IL-6, IL-1β, TNF-α, MMP-9, CD11B and GFAP were semiquantitatively analyzed by rtPCR of the penumbra.

**Results:**

24 hrs after reperfusion, rats were neurologically tested, euthanized and infarct volumes determined. Solulin significantly reduced mean total (p = 0.001), cortical (p = 0.002), and basal ganglia (p = 0.036) infarct volumes. Hippocampal infarct volumes (p = 0.191) were not significantly affected. Solulin significantly downregulated the expression of IL-1β (79%; p < 0.001), TNF-α (59%; p = 0.001), IL-6 (47%; p = 0.04), and CD11B (49%; p = 0.001) in the infarcted cortex compared to controls.

**Conclusions:**

Solulin reduced mean total, cortical and basal ganglia infarct volumes and regulated a subset of cytokines and proteases after tMCAO suggesting the potency of this compound for therapeutic interventions.

## Background

Stroke is a major cause of morbidity and mortality in the Western civilization. Roughly 60% of ischemic strokes are attributable to large-artery occlusion by thrombembolism. Complete absence of perfusion results in irreversible brain damage and neuronal loss in the stroke core. However, the surrounding penumbra contains functionally impaired, yet reversibly damaged neurons which are potentially salvageable. The goal in modern stroke therapy, therefore, is to protect the penumbra. To date, the only approved drug for lysis therapy is recombinant tissue plasminogen activator (rtPA) which has shown significant benefit in patient outcome when given up to 4.5 hours of onset. Less than 10% of all acute stroke patients are eligible for this treatment. No clinical trial has been able to demonstrate clear beneficial effects in respect to improvement of short- or long-term outcome after anticoagulatory therapy in acute ischemic stroke. Therefore, there is still a need for safe anticoagulatory substances with sufficient antithrombotic effectiveness and minimal risk of hemorrhagic side effects.

Thombomodulin (TM) is an endothelial cell transmembrane protein that acts as a thrombin receptor to modulate coagulation and fibrinolysis [1] and mediates anti-inflammatory effects [2]. Solulin (INN: sothrombomodulin alpha) is a recombinant soluble analogue of human TM consisting of its extracellular domains and distinguished by some point mutations to enhance its resistance against proteases and oxidation [3]. Most of TMs's/Solulin's known activities are dependent on binding of thrombin, the Solulin/thrombin complex activating Thrombin Activatable Fibrinolysis Inhibitor (TAFI) and, at higher concentrations, the serine protease activated protein C (APC) [4-6]. Activated APC with protein S as a co-factor attenuates the clotting cascade by digestion of activated clotting factors Va and VIIIa and prevents generation of thrombin and, finally, fibrin [7,8]. APC-mediated anticoagulant effects of Solulin have been implicated in its ability to restore cerebral flood flow and decrease infarct volume in a murine model of photothrombotic stroke [9]. In addition, TM displays a variety of anti-inflammatory and anti-apoptotic activities, mediated by APC and the lectin-like domain of TM [10]. The latter involves binding to and cleavage of HMGB1, a pro-inflammatory high mobility group box protein [2]. It also involves actions through pathways capable of dampening endothelial responses to proinflammatory stimuli [11,12].

In addition, anti-inflammatory and anti-apoptotic activities mediated by the lectin-like domain of TM are also discussed [11,12], which has been reported to bind and inhibit HMGB1, a pro-inflammatory high mobility group box protein [2].

In this study we analyzed whether Solulin, besides its known antithrombotic effects, exerts neuroprotective effects under transient ischemic conditions. Primary outcome parameter was infarct volume. Secondary outcome parameters were neurological outcome, mortality, hemorrhagic adverse events, and gene expression. A set of pro-inflammatory cytokines and proteases and microglial (CD11B) as well as astroglial (GFAP) markers were analyzed in the penumbra after 2 hours of tMCAO.

## Methods

### Study drug

Solulin was provided by PAION Deutschland GmbH (Aachen, Germany).

### Animals and transient middle cerebral artery occlusion (tMCAO) procedure

Research and animal care procedures were approved by the Review Board for the Care of Animal Subjects of the district government (LANUV (Landesamt für Natur, Umwelt und Verbraucherschutz, Northrhine-Westfalia, Germany)).

Male Sprague Dawley rats (250-280 g, Harlan Laboratories, Boxmeer, Netherlands; n = 10) were randomized to group 1 (n = 5) = control group without treatment or group 2 (n = 5) = treatment with 1 mg/kg body weight (BW) Solulin i.v. in normal saline (injection volume 5 ml/kg BW). Previous preclinical *in vivo *studies in rats yielded the best beneficial effect at doses from 0.1 to 1 mg/kg BW, which showed increasing anticoagulatory efficacy but no adverse events like hemorrhagic transformation that would be anticipated due to bleeding time prolongation at excessive doses. In this study, therefore, we decided to treat rats with a Solulin dose of 1 mg/kg BW.

Animals were held in macrolone cages (Ehret GmbH, Emmendingen, Germany) in a pathogen-free environment with food and water available ad *libitum*. They were subjected to 2 hrs of tMCAO using the intraluminal thread-occlusion technique as reported previously [13]. Animals were anesthetized with an intraperitoneal drug combination of 0.15 mg/kg BW Medetomidin [1 mg/ml] (Domitor^®^, Pfizer GmbH, Berlin, Germany), 2 mg/kg BW Midazolam [5 mg/ml] (Ratiopharm GmbH, Ulm, Germany) and 0.005 mg/kg BW Fentanyl [0.05 mg/ml] (JANSSEN-CILAG GmbH, Neuss, Germany) and maintained by an hourly intramuscular applicaton of 0.1-0.15 ml of the drug combination while breathing spontaneously. A small inguinal incision was made and after dissection of the left femoral artery and vein, polyethylene catheters were introduced for arterial blood gas measurements and intravenous drug application. ECG-needle electrodes were placed for continuous heart rate monitoring.

The left common carotid artery (CCA), internal carotid artery (ICA) and external carotid artery (ECA) were exposed through a midline neck incision. ECA and CCA were ligated proximally and the vagus nerve carefully dissected from the ICA. A 0.1% poly-L-lysine [14] coated 3-0 monofilament nylon suture (Hugo Sachs Elektronik, Harvard Apparatus, GmbH, March-Hugstetten, Germany) of 5 cm length was introduced through the distal CCA into the ICA and advanced until a resistance was felt indicating the bifurcation of anterior and middle cerebral artery, thus sufficiently occluding the MCA. Body temperature was maintained at 37-37.5°C with a heating pad (Fine Science Tools GmbH, Heidelberg, Germany) during the entire surgical procedure. To prevent bleeding, the exposed vessels were carefully ligated and a suture was tightened around the filament. The neck incision was closed aseptically, and animals were returned to their heated cages. Vehicle (5 ml/kg BW normal saline) or Solulin (1 mg/kg BW) in normal saline was applied i.v. by bolus injection 110 min after MCAO, i.e. 10 min before reperfusion (thread removal). Animals underwent neurological and behavioral scoring shortly before they were euthanized 24 hrs after tMCAO.

To assure appropriate MCA occlusion, regional cerebral blood perfusion (rCBF) over the left MCA was performed by laser-Doppler flowmetry (LDF). Through a small midline incision a 2-mm non-invasive laser-Doppler probe (PeriFlux System 5000, Type PF 5001, Perimed, Sweden) was placed on the animal's skull approximately 1-3 mm posterior to the bregma and 2-4 mm lateral to the midline. Baseline measurements were taken 10-15 minutes before tMCAO. Compared to baseline LDF showed a reduction of rCBF by at least 60% after MCAO [15]. There were no significant group differences.

### TTC-staining and measurement of infarct volume

Prior to brain dissection and mRNA extraction animals were deeply anesthetized and perfused transcardially with normal saline (0.9%) to wash out remaining circulating blood cells within the brain. Rat brains were then removed immediately, cut into 2 mm thick coronal sections using a rat brain matrix (Plastics One Inc., Roanoke, VA, USA) and stained in a 2% solution of 2,3,5-triphenyltetrazolium chloride (TTC) in normal saline (Bederson *et al*, 1986) at 37°C for 15 minutes. Vital tissue stained deeply red while infarcted tissue did not stain. Sections were then cryo-preserved. Images of the TTC-stained sections were acquired (Sony DXC 390P, Sony Germany GmbH). Total infarct volume as well as separate assessment of cortex, basal ganglia and hippocampal infarct areas were determined by image analysis software (Optimas 6.5, Adept Electronic Solutions Pty Ltd., Sydney, Australia). Total infarct volume was calculated by a blinded investigator by adding all cross-sectional areas multiplied by 2 mm (thickness of sections). Edema correction of infarct volume was achieved using the following equation [16]:

$${\text{V}}_{\text{edi}}={\text{V}}_{\text{infarct}}*\left(1-\left({\text{V}}_{\text{ipsi}}-{\text{V}}_{\text{contra}}\right)∕{\text{V}}_{\text{contra}}\right)$$

V _infarct _= Volume infarct, V _ipsi _= Volume ipsilateral hemisphere, V _contra _= Volume contralateral hemisphere, V _edi _= Volume edema corrected infarct

### Assessment of neurological deficits

Before euthanization 24 hrs after tMCAO, neurological testing was performed with a 6-point scale [17]. Assessment was executed as follows:

5 = normal motor function, no neurological deficit, 4 = flexion of torso and contralateral forelimb when lifted by the tail, 3 = decreased resistance to lateral push without circling, 2 = circling to the contralateral side against resistance when tugged by the tail on a flat surface, 1 = circling spontaneously to the contralateral side, 0 = no spontaneous motor activity, loss of walking or righting reflex.

### RNA isolation

On the basis of the TTC-staining results, total RNA was isolated from cerebral cortex of the adjacent penumbra regions of the infarcted hemispheres and the corresponding regions of the contralateral hemispheres from 3 consecutive coronal sections of each brain (sections 2,3 and 4) with NucleoSpin^®^RNA II (MACHEREY-NAGEL GmbH & Co. KG, Düren, Germany) according to the manufacturers' instructions.

Briefly, brain tissue was homogenized using Precellys Keramik-Kit (PEQLAB Biotechnologie GMBH, Erlangen, Germany) at 5000 × g for 15 s, cells were lysated with β-mercaptoethanol, filtrated by centrifugation, washed in 70% ethanol, DNA was digested, RNA purified in several washing steps and eluted in RNAse and DNAse free ultra-pure water (Invitrogen GmbH, Darmstadt, Germany).

### Gene expression

RNA concentration and purity were assessed using OD260/OD280 ratios (NanoDrop1000, PEQLAB Biotechnologie GmbH, Erlangen, Germany) and gel electrophoresis under denaturation conditions. Reverse transcription reactions were performed using M-MLV RT-kit (Invitrogen GmbH, Darmstadt, Germany) and random hexanucleotide primers. For gene expression analysis, we studied transcription levels of following factors: IL-6 (interleukin-6), IL-1β (interleukin-1β), TNF-α (tumor necrosis factor-α), MMP-9 (matrix metalloproteinase-9), CD11B and GFAP (glial fibrillary acidic protein) (all primers: Invitrogen GmbH, Darmstadt, Germany). All primers were designed with free access software (Primer3, Simgene.com). Primer sequences are listed in table 1. Semiquantitative rtPCR was performed. The amplified rtPCR products (10 μl) were electrophoretically separated on 1.5% agarose gels and stained with ethidium bromide. Real-time rtPCR (RT-rtPCR) reactions were carried out in a mixture consisting of 2 μl cDNA, 2 μl RNAse-free water (Invitrogen GmbH, Darmstadt, Germany), 5 μl 2×Sensi Mix × Plus SYBR & Fluorescein Kit (Quantace, Bioline GmbH, Luckenwalde, Germany) and 0.5 μl primers (10 pmol/μl). Reactions were conducted in standard tubes using the MyiQ RT-rtPCR detection system (Bio-Rad Laboratories, Munich, Germany) under the following conditions: 10 min enzyme activation at 95°C, 40 cycles of 15 s denaturation at 95°C, 30 s annealing at individual temperatures, 30 s amplification at 72°C, and 5 s fluorescence measurement at 72°C. External standard curves were generated by several fold dilutions of the target genes. The concentrations of the target genes were calculated by comparing Ct values in each sample with Ct values of the internal standard curve. Melting curve analysis and gel electrophoresis evaluation of the RT-rtPCR products were routinely performed to determine the specificity of the RT-rtPCR reaction. Gene expressions were normalized to the mean expression values of the housekeeping genes HPRT (hypoxanthine-guanine-phosphoribosyltransferase) and cyclophilin A.

**Table 1:** Oligonucleotide primers for RT-rtPCR

Gene	Sense/Antisense	Product size
HPRT	s 5'-GGTCCATTCCTATGACTGTAGATTTT-3' as 5'-CAATCAAGACGTTCTTTCCAGTT- 3'	125 bp
IL-6	s 5'-ACAGTGCATCATCGCTGTTC-3' as 5'-CCGGAGAGGAGACTTCACAG-3'	160 bp
IL-1β	s 5'-CTGTGACTCGTGGGATGATG-3' as 5'-GGGATTTTGTCGTTGCTTGT-3'	209 bp
TNF-α	s 5'-CTCCCAGAAAAGCAAGCAAC-3' as 5'-CGAGCAGGAATGAGAAGAGG-3'	209 bp
MMP-9	s 5'-GTCCGGTTTCAGCATGTTTT-3' as 5'-CCACCGAGCTATCCACTCAT-3'	158 bp
CD11B	s 5'-TTACCGGACTGTGTGGACAA-3' as 5'-AGTCTCCCACCACCAAAGTG-3'	239 bp
GFAP	s 5'-AGAAAACCGCATCACCATT-3' as 5'-GCACACCTCACATCACATCC-3'	187 bp

### Statistics

Sample size for the primary outcome parameter infarct volume was calculated. From prior experiments mean infarct volumes were expected around 300 mm^3 ^± 35 mm^3 ^with an anticipated 30% reduction of infarct volumes in the Solulin-treated cohort. The power was set to β = 0.8 and the significance level to α = 0.05 yielding a calculated sample size of four animals per group. In this study five animals per group were included. Power calculation was performed using nQuery Advisor^® ^Version 7.0 (Statistical Solutions, Saugus, Massachusetts, USA).

Edema corrected infarct volumes, and edema corrected mean cortical, basal ganglia and hippocampal infarct volumes were evaluated by two-way ANOVA. Multiple comparisons (heart rate, blood gas measurements and body temperature) were evaluated using an unpaired t-test. Neuroscores and gene expression data were evaluated by non-parametric Mann-Whitney-U-test with SPSS software version 17.0 (SPSS Inc., Chicago, IL, USA). All data are given as arithmetic means ± SEM (standard error of the means), or as means ± SD (standard deviation). P ≤ 0.05 was considered statistically significant. GraphPad PRISM^® ^(GraphPad Software Inc., La Jolla, CA, USA) was used to generate the figures.

## Results

### Edema corrected Infarct volumes

Edema corrected total infarct volumes were assessed, as well as infarct volumes separated for cortex, basal ganglia and hippocampus.

Brain edema formation in each group was determined indirectly by use of the above mentioned formula [16]. Comparison of both groups revealed no significant difference in the extent of brain edema (p > 0.05).

**Figure 1:**
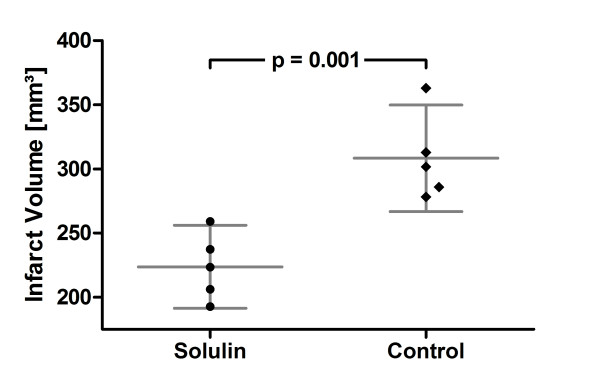
**Edema corrected mean total infarct volumes after 2 h of tMCAO 24 h post reperfusion**. Edema corrected infarct volumes (mm^3^) of Solulin-treated (left; n = 5) and vehicle-treated animals (right; n = 5). Significant reduction of infarct volumes in the Solulin-treated group (p = 0.001). Values are given as means ± SD.

**Figure 2:**
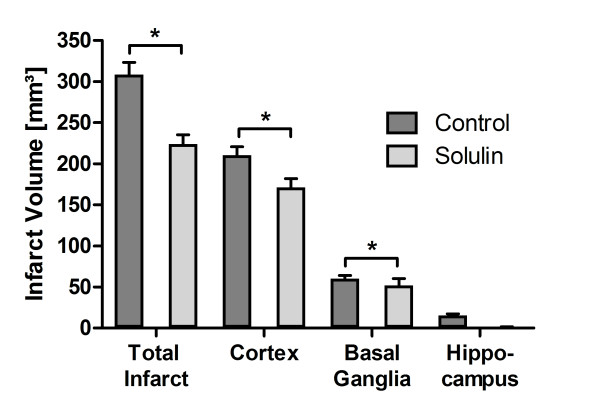
**Edema corrected mean cortical, basal ganglia and hippocampal infarct volumes after 2 h of tMCAO 24 h post reperfusion**. A significant reduction of cortical (p = 0.002) and basal ganglia (p = 0.036) infarct volumes was achieved. Hippocampal infarct volumes were nominally but not statistically significantly reduced. Values are given as means ± SEM.

A significant 27.4% reduction in mean total infarct volumes was achieved in Solulin-treated compared to vehicle-treated animals after tMCAO (Figure 1) (mean ± SD; controls, 308.33 ± 33.41 mm^3^, n = 5; Solulin, 223.72 ± 26 mm^3^, n = 5; p = 0.001). Similarly, significant reductions of cortical (19%) (mean ± SEM; controls, 210.44 ± 10.21 mm^3^; Solulin, 171.21 ± 10.52 mm^3^; p = 0.002) and basal ganglia infarct volumes (12.6%) (controls, 60.33 ± 3.66 mm^3^; Solulin, 52.08 ± 8.12 mm^3^; p = 0.036) could be detected. Hippocampal infarct sizes were reduced (controls, 15.43 ± 1.94 mm^3^; Solulin, 0.75 ± 0.75 mm^3^; p < 0.191) (Figure 2). However, this effect was not statistically significant. These data suggest that Solulin has a beneficial effect on the primary outcome parameter with marked reduction of infarct volumes.

No hemorrhagic transformation was observed in either group.

### Monitoring

Heart rate and body temperature did not differ between the groups. All blood gas measurements (pH, pO_2_, pCO_2_, cNa^+ ^or cK^+^) before and during ischemia or after reperfusion were comparable between the groups.

### Neurological deficit

Neurological deficits in both groups were equally distributed. Four animals had a score of 2 (circling against resistance) and one animal had a score of 1 (spontaneous circling) in each group, respectively.

### Quantitative gene expression

**Figure 3:**
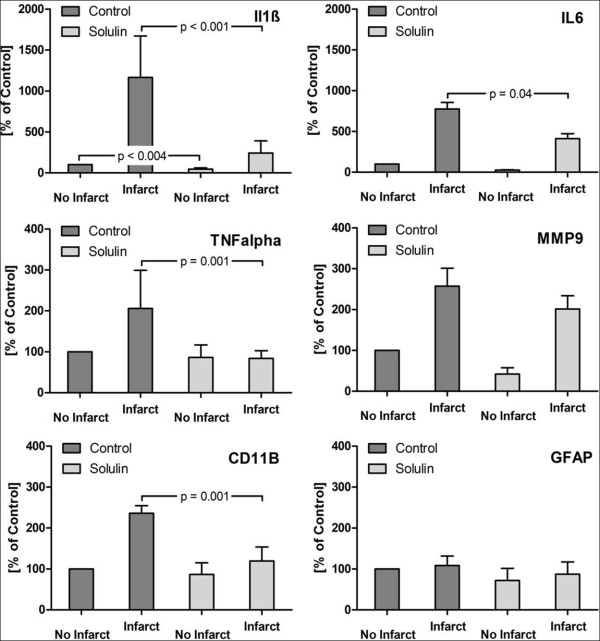
**Gene expression of IL-1β, IL-6, TNF-α, CD11B, MMP-9 and GFAP after 2 h of tMCAO 24 h post reperfusion**. Gene expression of IL-1β, IL-6, TNF-α, CD11B, MMP-9 and GFAP of 3 consecutive coronal sections of TTC-stained hemispheres. The contralateral non-infarcted hemispheres of the vehicle-treated group acted as internal controls. Values of these hemispheres were therefore set to 100%. All other values (infarcted hemisphere vehicle-treated group, infarcted and non-infarcted hemisphere of the Solulin cohort) were expressed as percentages of the latter. RT-PCR values were normalized to the housekeeping genes HPRT and cyclophilin A. All mRNA levels are given as means ± SEM.

HPRT and cyclophilin A served as a housekeeping gene to normalize gene expressions of brain tissue samples [18]. Gene expressions were semiquantitatively measured by RT-rtPCR using SYBR-Green (Quantace, Bioline GmbH, Luckenwalde, Germany) as intercalating dye. This technique is frequently used to determine gene expression levels. Values of the non-infarcted hemisphere of the vehicle-treated group were set to 100%. All other values (infarcted brain half vehicle-treated group, infarcted and non-infarcted hemisphere of the Solulin cohort) were expressed as percentages of the latter (Figure 3, table 2).

**Table 2:** Gene expression of IL-1β, IL-6, TNF-α, CD11B, MMP-9 and GFAP after 2 h of tMCAO and 24 h post-reperfusion

	Vehicle		Solulin	
Gene	Contralateral [%]	Ipsilateral [%]	Contralateral [%]	Ipsilateral [%]
IL-1β	100	1165.5 ± 1522.1	46.6 ± 42.8	242.7 ± 443.4
IL-6	100	775.2 * ± *724.4	26.1 ± 22.2	411.7 ± 550.6
TNF-α	100	206.2 ± 278.9	86.1 ± 92.4	83.9 ± 56.9
MMP-9	100	257.6 ± 131.1	41.9 ± 46.5	201.6 ± 97.1
CD11B	100	236.2 ± 56.1	86.6 ± 85.5	119.6 ± 102.8
GFAP	100	108.4 ± 69.5	71.7 ± 89.7	87.1 ± 89.7

Gene expression of  three consecutive coronal sections of TTC-stained hemispheres. The contralateral non-infarcted hemispheres of the vehicle-treated group acted as internal controls. Values of these hemispheres were therefore set to 100%. All other values (infarcted hemisphere vehicle-treated group, infarcted and non-infarcted hemisphere of the Solulin cohort) were expressed as percentages of the latter. RT-PCR values were normalized to the housekeeping gene HPRT and cyclophilin A. All mRNA levels are given as means ± SD.

A significant downregulation of IL-1β (79%; p < 0.001), TNF-α (59%; p = 0.001), IL-6 (47%; p = 0.04), and CD11B (49%; p = 0.001) was found in Solulin-treated infarcted hemispheres compared to untreated infarcted brain halves. Also MMP-9 (22%; p = 0.34) was nominally reduced, but the difference was not statistically significant. In the non-infarcted Solulin-treated hemispheres compared to untreated non-infarcted brain halves, gene expression was significantly lower for IL-1β (57%; p < 0.004) but not for TNF-α (p = 0.61), IL-6 (p = 0.063), MMP-9 (p = 0.11) and CD11B (p = 0.34).

No significant difference could be detected in GFAP (glial fibrillary acidic protein) expression neither between injured (p = 0.094) nor between non-injured hemispheres (p = 0.19) in both groups.

## Discussion

Solulin administration caused a down-regulation of the expression of inflammatory cytokines in the penumbra. In particular, TNF-α, IL-1β, and IL-6 were regulated. In addition, we observed that the expression of CD11B, a marker for microglia/macrophage activation, was also significantly reduced in Solulin-treated ischemic animals compared to untreated infarcted controls. GFAP, an astroglia marker, and MMP-9 were not regulated by Solulin. Solulin achieved a significant reduction in mean total infarct volumes (27.4%) with effective protection of both cerebral cortex (19%) and basal ganglia (12.6%). The overall reduction of infarct volumes does not suffice to explain the disparate decreases in expression of the various pro-inflammatory cytokines (IL 1β, 79%; TNF-α, 59%; IL-6, 47%).

Cerebral ischemia triggers a pathogenic cascade in which microglia are locally attracted and activated and express growth factors, chemokines, and regulatory inflammatory cytokines as mediators that attract mononuclear cells and granulocytes which further damage the ischemic brain tissue and its penumbra [19]. The mainly proinflammatory cytokines TNF-α and IL-1β may induce migration of neutrophils and macrophages into the CNS [20]. The up-regulation of both cytokines has often been demonstrated in transient and permanent MCAO which suggests that these cytokines play an important role in the inflammatory response associated with focal ischemia [20,21].

TNF-α serves as a marker for activation of microglia and macrophages during cerebral ischemia and other inflammatory reactions as reported previously [22] and appears to mediate cell death. Consistently, it was demonstrated that the administration of TNF-α antibodies or TNF-α binding receptors led to a reduction of infarct volumes in experimental animal stroke models [23,24]. Other studies, however, emphasize the neuroprotective potency of TNF-α with a role during repair and regeneration of brain tissue [25], making TNF-α another example of the time-dependent duality of certain inflammatory molecules, being destructive at the beginning while inducing an isochronic coordination of protective mechanisms. IL-1β expression, like TNF-α, seems to promote infarction progression in animal models of ischemic stroke [25]. Clausen and collaborators demonstrated that IL-1β mRNA and TNF-α mRNA, and TNF-α protein are produced by CD11b+ microglia/macrophages in the penumbra as well as the core of brain infarction [25]. CD11B is also expressed by immature dendritic cells of probably myeloid origin during neuroinflammation after brain infarction which may be associated with phagocytosis [26]. Its downregulation might involve a decreased degradation reaction by dendritic cells and a decreased activation of microglia and macrophages post-infarction, in turn leading to a decreased expression of TNF-α and IL-1β. IL-6 mRNA is produced during inflammatory processes of the CNS [27] and is up-regulated after pMCAO in rats [28]; however, its role in both acute and chronic brain damage is still unclear [29]. Whether its up-regulation is indicative of neuronal damage or neural regeneration is still uncertain [30].

There was no difference in expression of glial fibrillary acidic protein (GFAP), neither in infarcted nor in the unaffected hemispheres of both Solulin and control rats. GFAP is the principal intermediate filament in mature astrocytes and serves as a marker for reactive astrogliosis [30,31] after nervous tissue injury. Since tissue evaluation was done 24 hrs after tMCAO, the postoperative time span appears too short for a significant activation/proliferation of astrocytes. Thus, an increased GFAP-expression was not anticipated in our study.

Matrix metalloproteinases (MMP) are proteases related to extracellular matrix breakdown [32] inducing neuronal damage, blood-brain-barrier (BBB) disruption and rarely intracerebral hemorrhage. Degradation of the basal lamina and tight junctions of cerebral blood vessels leads to BBB disruption followed by formation of cytotoxic as well as vasogenic brain edema and consecutive apoptosis of neurons and oligodendrocytes [33]. MMPs might also play a role in abetting neutrophil and macrophage invasion [34]. MMP-9 is not expressed in the CNS but seems to invade the brain together with leukocyte infiltration induced by focal ischemia [35,36]. Together with platelet-derived growth factor CC [9], MMP-9 has been involved in BBB breakdown after stroke [35,37].

As cerebral reperfusion in the current study was produced by removal of the occluding thread, it appears unlikely that anticoagulation is central to Solulin's ability to reduce infarct volumes. Rather, its anti-inflammatory and anti-apoptotic activities, mediated by its lectin-like domain [11,12] and/or corresponding effects of APC [38] seem to be involved. Antibodies against HMGB1 have been found to ameliorate brain infarction induced by transient ischemia in rats, with inhibition of the expression of TNFα, but practically no effect on cerebral blood flow [20].

Nonetheless, there may also be a role for anticoagulation, as restoring blood flow may give rise to downstream displacement of material formed around the occluding suture. Solulin might also act to restore blood flow in the dependent microvasculature, which is likely to get clogged to some extent during the ischemia period and will not readily re-open once the occluding thread has been removed. This possibility is supported by the observation that Solulin, in the mouse photothrombotic model, was able to restore blood flow when administered 30 or 60 min post-occlusion [9]. From a different perspective, anti-inflammatory effects of Solulin may contribute to its beneficial effects in the mouse photothrombotic model: When administered 60 min after photothrombotic occlusion, Solulin only transiently restored blood flow, but was still able to cause a significant reduction of infarct size 24 hrs later [9]. Thus, anti-inflammatory and anticoagulant (profibrinolytic) effects of Solulin may act in concert. As inflammation is a powerful procoagulant/antifibrinolytic force [39], this profile would make Solulin a promising tool in the setting of acute cerebral ischemia.

## Conclusion

In response to the treatment with the recombinant soluble analogue of the human transmembrane protein thrombomodulin, Solulin, we could clearly demonstrate a significant reduction in mean total infarct volumes as well as of cortical and basal ganglia infarct volumes in a model of transient focal cerebral ischemia. We could further show that Solulin abolishes the induction of TNF-α, IL-1β, IL-6, CD11B as well as of MMP-9 in the penumbra. The underlying mechanisms of action are not yet fully understood, but seem to involve anti-inflammatory effects, possibly aided by activation of protein C and anticoagulant (profibrinolytic) effects. Requisite to understanding how this promising substance brings about the profound reduction infarct volumes, future neurocognitive studies are indicated.

## Competing interests

KUP is an employee of PAION Deutschland GmbH, Aachen. All other authors declare that they have no competing interests.

## Authors' contributions

YMR and JD conducted the experimental laboratory work. YMR and MC performed the statistical analysis and drafted the manuscript. KUP and RR participated in the study design and coordination and helped to draft the manuscript. MK, CB, AF and DW helped to perform the study and draft the manuscript. All authors read and approved the final manuscript.
